# Anosmia but Not Ageusia as a COVID-19-Related Symptom among Cancer Patients—First Results from the PAPESCO-19 Cohort Study

**DOI:** 10.3390/cancers13143389

**Published:** 2021-07-06

**Authors:** Ke Zhou, Audrey Blanc-Lapierre, Valérie Seegers, Michèle Boisdron-Celle, Frédéric Bigot, Marianne Bourdon, Hakim Mahammedi, Aurélien Lambert, Mario Campone, Thierry Conroy, Frédérique Penault-Llorca, Martine M. Bellanger, Jean-Luc Raoul

**Affiliations:** 1 Department of Human and Social Sciences, Institut de Cancérologie de l’Ouest (ICO), 44805 Saint-Herblain, France; marianne.bourdon@ico.unicancer.fr (M.B.); martine.bellanger@ico.unicancer.fr (M.M.B.); 2 Department of Biostatistic, Institut de Cancérologie de l’Ouest, 44805 Saint-Herblain, France; audrey.blanc-lapierre@ico.unicancer.fr (A.B.-L.); valerie.seegers@ico.unicancer.fr (V.S.); 3 Department of Biopathology, Institut de Cancérologie de l’Ouest, 49055 Angers, France; michele.boisdron@ico.unicancer.fr; 4 Department of Medical Oncology, Institut de Cancérologie de l’Ouest, 49055 Angers, France; frederic.bigot@ico.unicancer.fr; 5 Research Unit UMR INSERM 1246 SPHERE, Universités de Nantes et Tours, 44035 Nantes, France; 6 Department of Medical Oncology, Centre Jean Perrin, 63011 Clermont-Ferrand, France; hakim.mahammedi@clermont.unicancer.fr; 7 Department of Medical Oncology, Institut de Cancérologie de Lorraine, 54511 Vandoeuvre-lès-Nancy, France; a.lambert@nancy.unicancer.fr (A.L.); t.conroy@nancy.unicancer.fr (T.C.); 8 Department of Medical Oncology, Institut de Cancérologie de l’Ouest, 44805 Saint-Herblain, France; mario.campone@ico.unicancer.fr (M.C.); jean-luc.raoul@ico.unicancer.fr (J.-L.R.); 9 Department of Biopathology and INSERM U1240, Centre Jean Perrin, 63011 Clermont-Ferrand, France; frederique.penault-llorca@clermont.unicancer.fr; 10 Department of Social Sciences, EHESP School of Public Health, 35043 Rennes, France

**Keywords:** cancer, COVID-19, symptoms, healthcare workers, anosmia, dysgeusia, ageusia, France, serological test, RT-PCR

## Abstract

**Simple Summary:**

COVID-19 has some clinical manifestations that are similar to the side effects of cancer treatments such that cancer patients may fail to distinguish COVID-19 symptoms from those of their treatments. The PAPESCO-19 study investigated 13 COVID-19 symptoms and confirmed that in combination with anorexia, fever, headache, and rhinorrhea, anosmia has a strong association with COVID-19 for cancer patients while dysgeusia/ageusia does not.

**Abstract:**

**Background:** Cancer patients may fail to distinguish COVID-19 symptoms such as anosmia, dysgeusia/ageusia, anorexia, headache, and fatigue, which are frequent after cancer treatments. We aimed to identify symptoms associated with COVID-19 and to assess the strength of their association in cancer and cancer-free populations. **Methods:** The multicenter cohort study PAPESCO-19 included 878 cancer patients and 940 healthcare workers (HCWs). At baseline and quarterly thereafter, they reported the presence or absence of 13 COVID-19 symptoms observed over 3 months and the results of routine screening RT-PCR, and they were systematically tested for SARS-CoV-2-specific antibodies. We identified the symptom combinations significantly associated with COVID-19. **Results:** Eight percent of cancer patients were COVID-19 positive, and 32% were symptomatic. Among the HCWs, these proportions were 9.5 and 52%, respectively. Anosmia, anorexia, fever, headache, and rhinorrhea together accurately discriminated (c-statistic = 0.7027) COVID-19 cases from cancer patients. Anosmia, dysgeusia/ageusia, muscle pain, intense fatigue, headache, and chest pain better discriminated (c-statistic = 0.8830) COVID-19 cases among the HCWs. Anosmia had the strongest association in both the cancer patients (OR = 7.48, 95% CI: 2.96–18.89) and HCWs (OR = 5.71, 95% CI: 2.21–14.75). **Conclusions:** COVID-19 symptoms and their diagnostic performance differ in the cancer patients and HCWs. Anosmia is associated with COVID-19 in cancer patients, while dysgeusia/ageusia is not. Cancer patients deserve tailored preventive measures due to their particular COVID-19 symptom pattern.

## 1. Introduction

Because of the worldwide spread of COVID-19, patients with cancer might be more susceptible to SARS-CoV-2 infection [1]. Those who have had hematological malignancy, lung or metastatic cancer or who have undergone surgery or immunotherapy are at an even higher risk of severe symptoms, admission to an intensive care unit, mechanical ventilation or death [2,3,4,5]. Clinical factors, such as advanced cancer stage and subtype, may be associated with poorer COVID-19 outcomes [1,2,5] alongside age, sex, and comorbidities [5,6,7,8]. As a result, cancer patients deserve particular attention when identifying the specific early symptoms of COVID-19 for further diagnosis.

The combination of specific symptoms, including fever and persistent cough and more surprisingly, anosmia (loss of smell) and dysgeusia/ageusia (distortion/loss of the sense of taste) make it possible to diagnose individuals with COVID-19 [9,10,11,12,13,14,15,16,17]. However, smell and taste alteration are also frequently observed in cancer patients during treatments [18,19,20,21,22,23]. U.K. clinicians have raised concerns about this overlap, recommending that oncology patients be carefully advised, and they have called for more evidence on the topic [24].

Studies reporting associations between seroprevalence and self-reported symptoms for healthcare workers (HCWs) found that fever, malaise, and fatigue were especially common alongside anosmia and ageusia [25,26]. However, little is known about HCWs from oncology departments and cancer centers despite the fact that they have been a under major strain during the pandemic [27,28].

In France, only one cross-sectional study reported significantly more frequent anosmia and ageusia in seropositive cancer patients and HCWs than in seronegative ones. This was demonstrated by analyzing factors associated with the seroprevalence of SARS-CoV-2 after the first lockdown among two small populations of seropositive patients and HCWs from a single cancer center in Burgundy [27]. Therefore, more research is needed to investigate the predictive values of COVID-19-related symptom patterns in patients undergoing cancer treatments.

We are conducting a French study involving two cohorts of cancer patients and HCWs, who were systematically tested for SARS-CoV-2 infection during the ongoing pandemic as described below. This paper aims to identify symptoms which may predict COVID-19 in cancer patients and HCW populations. Our findings may help guide COVID-19 diagnostic, screening, and prevention strategies for cancer patients. 

## 2. Materials and Methods

### 2.1. Study Design and Setting

We initiated a multicenter cohort study involving cancer patients and HCWs—*PAtients et PErsonnels de Santé des Centres de Lutte Contre le Cancer pendant l’épidémie de COvid-19* (PAPESCO-19)—at comprehensive cancer centers in three different French regions: the Nantes and Angers sites of the ICO Cancer Center (Western France); the Lorraine Cancer Center in Nancy (Eastern France); and the Jean Perrin Cancer Center in Clermont-Ferrand (Central France) [29]. These regions are of interest as the 2020 COVID-19 epidemic had different local impacts [30]. The PAPESCO-19 study consists of four work packages, each with a different focus: serological and clinical, public health, economic, and psychological. We plan to include 3500 individuals.

We collected previous COVID-19 tests and symptoms reported by participants when they enrolled at baseline, as explained below in Section 2.3 Data collection. We then prospectively examined participants’ COVID-19-related outcomes at quarterly intervals: 3, 6, 9, and 12 months.

Although the study has a broad scope and is ongoing, this analysis is based on the serological and clinical work package using data collected between the first participant’s enrollment on 17 June 2020, and the date of the first four-month report on 30 November. This data allowed the effects of two epidemic waves to be captured.

### 2.2. Participants

We included patients aged ≥18 years attending cancer centers as part of an ongoing active treatment (radiotherapy, surgery, immunotherapy or chemotherapy) or monitoring of treatment completed more than a year earlier. HCWs (nurses, clinicians, and other cancer center staff) enrolled voluntarily after being informed of the study via email and the cancer center intranet. Cancer patients and HCWs were recruited from each of the three regions. Although both cohorts within a site would have been exposed to the same spatio-temporal spread of COVID-19, they were not designed to be comparable as the two populations were not matched on the baseline characteristics of age, sex or comorbidities. 

Participants were eligible irrespective of whether they had presented with symptoms since start of the COVID-19 outbreak. The inclusion period was one year with follow-up visits planned every three months. All participants signed an informed consent form, and the study is being conducted in accordance with the Declaration of Helsinki. The Ethics Committee (CPP-IDF VIII, Boulogne-Billancourt) approved our study number 20.04.15 on 15 May 2020.

### 2.3. Data Collection

#### 2.3.1. Study Questionnaires

At baseline and follow-up, all participants completed questionnaires on sociodemographic and lifestyle characteristics and on COVID-19-related history, including exposure to infected people, self-reported COVID-19 symptoms (detailed below), results of previous RT-PCR tests, and ambulatory care use. For cancer patients, baseline demographic data (age and sex), cancer history, and clinical details were recorded in electronic case report forms (eCRFs). For the HCWs, we collected demographic data (age and sex), job role, and occupation type (e.g., physician, nurse, assistant nurse, or pharmacist) as well as clinical data (self-reported body weight and height, comorbidities, and comedications) based on a questionnaire.

#### 2.3.2. Blood Samples, Serological Tests and Routine RT-PCR Reported

We used the lateral flow immunoassay (LFIA) NG-Test^®^ IgG–IgM COVID-19 (NG Biotech Laboratoires, Guipry-Messac, France): “[an] immune colloidal technique intended for the qualitative detection of IgG and IgM antibodies against the SARS-CoV-2 nucleoprotein in serum or plasma” [31]. Studies comparing the clinical performance of LFIA NG-Test^®^ with one or more automated immunoassays—the enzyme-linked immunosorbent assays (ELISA IgG/IgA) is the gold standard—and SARS-CoV-2 chemiluminescence enzyme immunoassays (CLIA–IgG) found similar results. The performance of LFIA tests showed a sensitivity to SARS-CoV-2-specific IgG of about 100% (95% CI: 95.5–100.0%) for the late disease stage (14 days after symptom onset. The specificity during the same late disease stage was 95.3% (95% CI: 90.7–97.7%) for SARS-CoV-2-specific IgG and IgM [31,32,33,34]. 

Serological tests were performed in the clinical biology department of each cancer center. At baseline, they were able to detect SARS-CoV-2-specific IgG antibodies after an infection during the first epidemic wave [35].

Participants also reported the results of routine RT-PCR tests completed independently of this study as a result of symptoms or possible contacts.

#### 2.3.3. Reported Symptoms

At baseline, participants reported COVID-19-related symptoms from the beginning of the epidemic, including the first epidemic wave. During a follow-up, patients reported symptoms since the previous visit, which was before blood sampling was carried out. At each follow-up, the questionnaire included detailed, predefined symptoms (including onset and end dates), which the patient did not identify as being related to any treatment. The symptoms included were fever >38 °C, headache, anosmia, dysgeusia/ageusia, rhinorrhea, unusual cough, shortness of breath, muscle pain, intense fatigue, anorexia, red eyes (conjunctivitis), digestive disorders (diarrhea, vomiting, and abdominal pain), and chest pain [36,37]. Symptoms were not graded (NCI-CTCAE) as part of the study, but the vast majority were assumed to be mild (grade 1) because the cancer patients were instructed to seek hospital care in the event of moderate or severe symptoms.

### 2.4. COVID-19 Test Outcomes and Symptoms

Participants with at least one SARS-CoV-2-positive serological test or RT-PCR test result during the study were considered to have a COVID-19 positive outcome (COVID+), while participants with consistently negative test results throughout the study were considered to be uninfected (COVID–).

Participants were considered to be symptomatic if they reported any of the 13 listed symptoms at any point, which was in accordance with the definition of a symptomatic patient in the Infectious Diseases Society of America (IDSA)’s guidelines: “[having] at least one of the most common symptoms compatible with COVID-19” [38]. This approach was used by many previous studies [11,13,15,25]. Participants who did not report any of the 13 symptoms were considered asymptomatic [28,38]. 

We then analyzed the association between positive COVID-19 test results and reported symptoms to assess the cancer patient cohort and HCW cohort independently.

### 2.5. Statistical Analysis

We estimated the proportions of participants with positive serological tests or positive self-reported RT-PCR tests with a 95% confidence interval (CI). No statistical comparison was made between the two cohorts.

During univariate analysis, we estimated the proportion of participants who were symptomatic and used logistic regression to estimate the odds ratios (ORs) of the association between COVID-19 test outcomes and symptom presentation. We also estimated the sensitivity (Se), specificity (Sp), and OR for every symptom. All diagnostic performance indicators were reported with a 95% CI.

We further investigated the symptom pattern associated with a positive COVID-19 test in a multivariate model. Backward logistic regression was performed, starting with a full model that included the 13 symptoms with a variable entry and removal threshold of *p* = 0.20. None of these variables included missing data. We used the Akaike information criterion (AIC) for model selection. The model was developed independently in the patient and HCW cohorts.

We reported the models’ Se, Sp, and accuracy values. C-statistic was used to evaluate the models’ performance and discrimination ability [39,40].

To validate the final models, we stratified each cohort by sex, median age, and cancer features (for the cancer patient cohort), and combined two or more pertinent strata to perform the logistic regression if there were insufficient covariate variations in one stratum. We drew 75 and 25% of the samples at random from the cancer patient and HCW cohorts, respectively. To account for differences in epidemic exposure among the cancer centers, we grouped Nantes and Angers cancer patients as one subpopulation, and the Nancy and Clermont-Ferrand cancer patients as another. The same analysis was done for the HCWs.

Of note, we reported any missing data in the descriptive tables and excluded this missing data when calculating percentages. No imputation was made since the missing data were not included as covariates in the regression analysis. The Ennov Clinical^®^ system was used for data collection, and SAS^®^ 8.3 and STATA^®^ 14.2 were used for statistical analysis.

## 3. Results

### 3.1. Population Characteristics

Total enrollment was 878 cancer patients and 940 HCWs. The median age was 62 years (18–91) for the cancer patients and 40 for the HCWs (19–66). Women represented more than two-thirds of the cancer patients (69%) and three-quarters of the HCWs (81%). Forty-one percent (330/811) of the cancer patients and 13% (123/940) of the HCWs had at least one comorbidity. A public-facing role was observed in 42% (204/487) of the cancer patients and 81% (740/917) of the HCWs (Table 1 and Table S1).

Ninety percent (845/878) of cancer patients were undergoing cancer treatments of whom (433/878 = 55.6%) had metastatic cancer. Almost half (371/811 = 46%) had breast cancer, followed by uterine, endometrial, and cervical (86/811 = 11%). Among male cancer patients, prostate cancer was the most prevalent type (59/262 = 23%), followed by urological (56/262 = 21%). Fifty-nine percent (462/788) received chemotherapy as the last treatment before their inclusion in this study (Table 2).

### 3.2. COVID-19 Outcomes

Seventy cancer patients (8%, 95% CI: 6–10%) (Table 1) and 89 HCWs (9.5%, 95% CI: 8–12%) tested positive for COVID-19 (Table S1). Systematic serological tests detected COVID-19 cases in 6.7% (95% CI: 5–9%) of cancer patients (Table 1) and 7.8% (95% CI: 6%–10%) of HCWs (Table S1). Of one in five participants (16.7% of cancer patients and 23.5% of HCWs) were having routine RT-PCR screening; 3% (95% CI: 2–4%) of cancer patients tested positive (Table 1) and 5% (95% CI: 4–7%) of HCWs (Table S1).

There was a significantly higher proportion of COVID+ results among female cancer patients than male patients (*p* = 0.04), and among cancer patients being monitored than those undergoing treatment (*p* = 0.01). Furthermore, a significantly higher proportion of cancer patients with kidney failure tested positive for COVID-19 than did those without this condition (Table S2).

Eighteen cancer patients (2.1%) and four HCWs (0.4%) were hospitalized: one cancer patient was admitted to the intensive care unit, and another died as a result of COVID-19. Overall, 70 cancer patients had a COVID-19 infection, which was asymptomatic or mild in 74.3% of cases, moderate in 22.8%, and life-threatening in 2.8% with a mortality rate of 1.4%.

Among cancer patients, the highest proportion of COVID+ was observed in Nancy (9.3%) and the lowest in Clermont-Ferrand (6.3%) (Table S3), though no significant differences were observed between the centers (*p* = 0.27). Despite a distance of less than 100 km between the sites, the HCWs in Nantes had more than double the proportion of positive COVID-19 tests than those in Angers (11.7 vs. 5.0% respectively, *p* = 0.01) (Table S3).

### 3.3. Single Symptoms

A total of 282 (282/878 = 32%) cancer patients and 485 (485/940 = 52%) HCWs were symptomatic (Table 1). Among the COVID+ participants, 29 cancer patients (29/70 = 41%) and 8 HCWs (8/89 = 9%) were asymptomatic.

In cancer patients, we noted differences between centers in reported symptom prevalence, with 16.1% in Nancy (a badly affected Eastern region) and 46.3% in Nantes (a less-affected region, *p* < 0.001). Among the HCWs, we observed that after the 30–40-year age range, reported symptom prevalence decreased with increasing age (*p* = 0.004). Symptoms in HCWs were less prevalent in Angers than in Nantes (44.6 and 54.7%, respectively, *p* = 0.019) and overall differences among centers were significant (*p* = 0.018) (Table S3).

**Figure 1:**
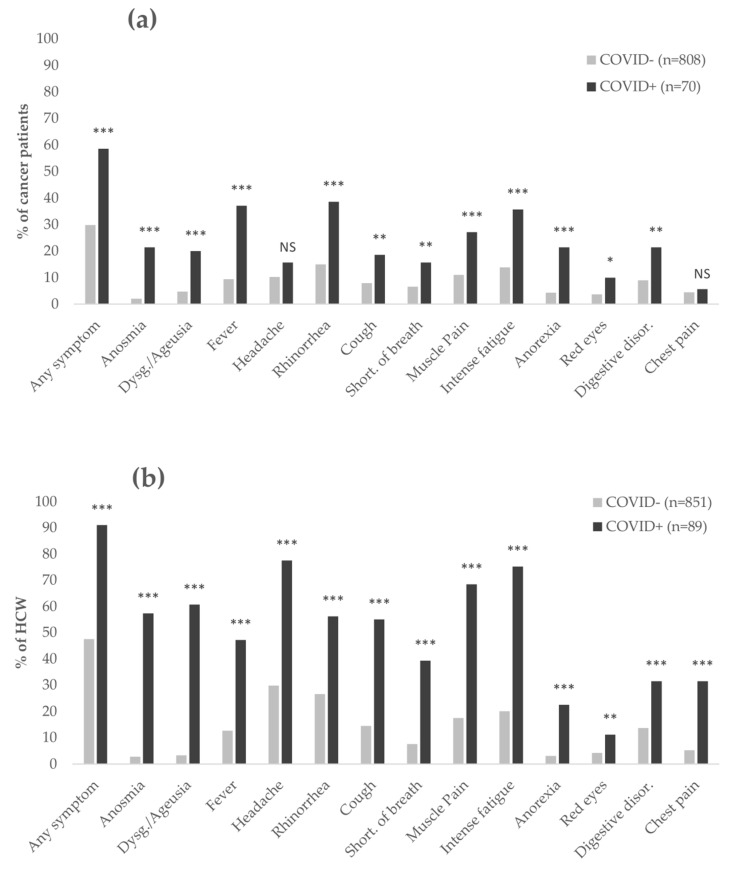
Bar chart, % of COVID+ and COVID- participants reporting the presence of each symptom. (**a**) Symptoms in cancer patients. (**b**) Symptoms in healthcare workers. Any symptom: having at least one of the symptoms; Anosmia: smell blindness. In the questionnaire, the description was “loss of smell”; Dysg. (Dysgeusia): distortion of the sense of taste. Ageusia: loss of the sense of taste. In the questionnaire, the description was “alteration or even loss of taste”; Rhinorrhea: free discharge of a thin nasal mucus fluid, runny nose; Short. of breath: shortness of breath; Anorexia: Eating disorder; Digestive disor. (Digestive disorders): diarrhea, vomiting, and abdominal pain; *p*-value: univariate logistic regression, NS: Non-Significant, * *p* < 0.05, ** *p* < 0.01, *** *p* < 0.001.

In a univariate analysis of the cancer patient cohort, all symptoms except headache and chest pain showed statistical significance against COVID-19 test outcomes (Figure 1a). All symptoms showed significance for the HCWs (Figure 1b).

**Figure 2:**
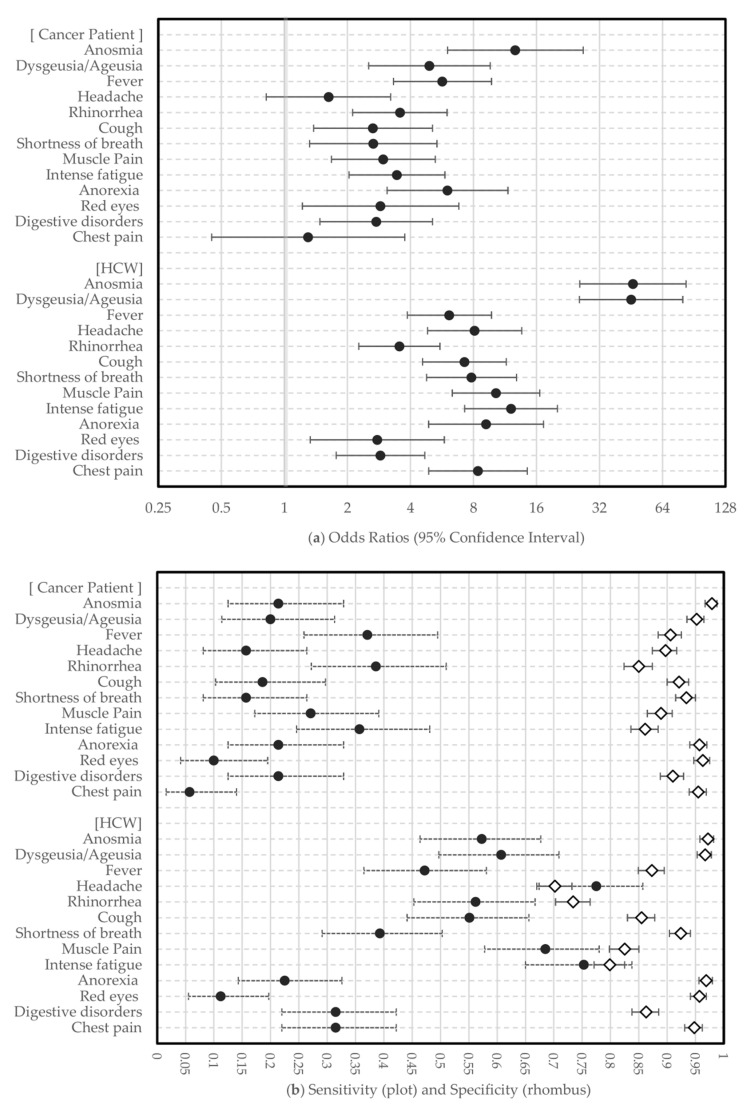
Forest plot. (**a**) The Odds Ratio (OR) of the association between each symptom and a positive COVID-19 test. (**b**) Sensitivity (Se) and specificity (Sp) of each symptom.

Figure 2a shows the OR of the association between each symptom and a positive COVID-19 test. Among cancer patients, anosmia had the highest OR (12.69, 95% CI: 6.02–26.76). However, dysgeusia/ageusia had a lower OR (4.93, 95% CI: 2.53–9.62) than fever (OR = 5.69, 95% CI: 3.32–9.76) and anorexia (OR = 6.02, 95% CI: 3.10–11.70). Among the HCWs, anosmia and dysgeusia/ageusia had similar ORs (46.25, 95% CI: 25.79–82.93; 45.35, 95% CI: 25.69–80.04, respectively), which were the highest of all of the symptoms.

The Se and Sp of each symptom are presented in Figure 2b. Anosmia was the most specific symptom for cancer patients (Sp = 0.98, 95% CI: 0.97–0.99). Anosmia and dysgeusia/ageusia were the most specific symptoms for the HCWs (anosmia Sp = 0.97, 95% CI: 0.96–0.98; dysgeusia/ageusia Sp = 0.97, 95% CI: 0.95–0.98). Rhinorrhea was the most sensitive symptom, though not very high, for the cancer patients (Se = 0.39, 95% CI: 0.27–0.51), and headache was the most sensitive HCW symptom (Se = 0.78, 95% CI: 0.67–0.86).

### 3.4. Combined Symptoms Predicting COVID-19

Table 3 reports the results of the backward variable selection logistic regression. For cancer patients, in the final model anosmia was the most significant symptom (OR = 7.48, 95% CI: 2.96–18.89, *p* < 0.001). Holding all other factors constant, a patient with anosmia has 7.5 times greater odds of having a positive test than a patient who did not report anosmia. Among the four other selected symptoms, anorexia, fever, and headache were the most significant (*p* < 0.05). Surprisingly, headache had an OR of less than 1. It is worth noting that dysgeusia/ageusia had not been selected as a significant predictor of COVID-19 positive test outcome among cancer patients.

For HCWs, anosmia and dysgeusia/ageusia were significant symptoms with the largest ORs for the COVID-19 positive test outcome (anosmia OR = 5.71, 95% CI: 2.21–14.75; dysgeusia/ageusia OR = 5.14, 95% CI: 2.01–13.14) in the final model. Muscle pain, intense fatigue, headache, and chest pain (*p* < 0.05) were also selected symptoms.

Overall, the selected symptoms better classified COVID-19 test outcomes for HCWs than for cancer patients (c-statistic = 0.8830 vs. 0.7027) (Table S4).

The equations used to estimate the probability of a COVID-19 positive test outcome are reported in Supplementary II.

### 3.5. Model Validation

In the cancer patient model, stratifying by sex, median age, and cancer-related variables did not influence the model’s validity given the very limited change in the c-statistics (Table S4). The same was observed for cancer treatment types, such as chemotherapy and immunotherapy. Less than 10% variation in the c-statistics was observed within the 75 and 25% samples. Stratifying by centers of inclusion resulted in smaller variation in performance measurements (Table S4).

In the HCW model, the c-statistics ranged from 0.7989 to 0.9241. The final model had relatively stable performance measurement values in all subpopulations (Table S4).

Finally, when comparing sub-datasets stratified with the same factors, the HCW model consistently performed better than the cancer patient model.

## 4. Discussion

Based on the PAPESCO-19 multicenter cohorts, the current study identified symptoms which could predict SARS-CoV-2 infection in 878 cancer patients and 940 oncology HCWs.

Proportions of COVID+ cases were similar; 8% for cancer patients and 9.5% for HCWs. Nevertheless, more cancer patients than HCWs had severe outcomes (hospitalized: 2.1 vs. 0.4%; ICU and death: 1 vs. 0). Symptoms considered to be associated with COVID-19 had a lower prevalence among cancer patients (32%) than among HCWs (52%). Almost all COVID+ HCWs (91%) were symptomatic, while approximately half (49%) of COVID+ cancer patients reported symptoms. In contrast, 5% of asymptomatic cancer patients and 2% of asymptomatic HCWs were COVID+.

For the cancer patients, one immediate finding was that a single symptom had a weak relationship with COVID-19 test results. It is worth noting that cancer patients usually suffer from different symptoms, so they possibly failed to notice the additional symptoms of SARS-CoV-2 infection. Nevertheless, combining several symptoms (anosmia, fever, headache, rhinorrhea, and anorexia) discriminated COVID-19 positivity fairly well. This was consistently seen within different sub-datasets, stratified by factors such as cancer treatment or type. Negative association with headache may serve as a differential diagnosis criterion and improve diagnostic accuracy. When assessed in combination with the other symptoms, a headache more likely suggests a condition other than COVID-19, whereas its absence raises the possibility of having COVID-19. Cancer patients deserve tailored preventive measures irrespective of the absence of symptoms.

For the HCWs, single symptoms had a strong association with COVID-19 test results. We identified several common symptom combinations validated in previous studies. These were anosmia, dysgeusia/ageusia, muscle pain, and intense fatigue [11,15]. Our study further included headache and chest pain, which differed from other symptoms in the previous studies’ models.

It is worth noting that, in PAPESCO-19, digestive symptoms, particularly diarrhea were not associated with COVID-19 positivity in contrast to other studies. However, in our population of cancer patients, diarrhea was reported in 21.4% of COVID+ cases and in 4% of COVID-; similarly for the HCWs, these figures were 31.5 and 13.7%, respectively. These data were in accordance with what has been observed in the literature, with gastrointestinal incidence (mainly diarrhea) ranging from 4 to 50% of cases [27,41].

Anosmia, as a single symptom or when combined with others, remained strongly associated with COVID-19 positivity. While dysgeusia was also found to be a good predictor for identifying COVID-19 among the HCWs (though less frequent than anosmia [9,10,11,12,13,14,15,16,17]), this was not the case for the cancer patients.

Our findings from the PAPESCO-19 study were strengthened by both the study period, which covered two main waves of the pandemic in France, and by the inclusion of different geographical locations having varying epidemic levels [30,42]. This diversity reinforced the robustness and extrapolability of our final models since symptom diagnostic performance depends on the prevalence of symptomatic and COVID+ cases. In addition, the design of our study enabled us to capture COVID-19 cases comprehensively with systematic testing of all participants irrespective of symptoms.

The overall COVID-19 prevalence we observed was, to a certain extent, comparable to a French survey that covered only the first wave, in which 7% of participants were COVID-19 positive [43]. There were significant regional differences in symptom prevalence and in the proportion of COVID+ cases among the HCWs, but not the cancer patients (Table S3). The effect of the cancer patients’ self-protective measures should be investigated in future studies.

Severe COVID-19 cases might be underrepresented in our study. Only 1.2% of participants were hospitalized due to the infection and only one death was reported compared with 23 cancer-related deaths. It was more likely that individuals with SARS-CoV-2 infections had been admitted to specialized COVID-19 hospitals and did not attend cancer centers during the PAPESCO-19 recruitment period. 

Self-reporting bias may have affected the collected data, especially the self-declared symptoms, which may have been overreported or underreported as was observed in previous studies [11,44]. In addition, recall bias was inevitable due to the gap between the participant’s inclusion date (baseline questionnaire) and the first wave of the pandemic in France (mid-March 2020) [45].

Our estimates of COVID+ cases are partially based on the LFIA NG-Test^®^, and we acknowledge that the reliability of COVID-19 serological tests is a function of the humoral immune response pattern. However, studies showed that antibody titres were negative in about 5% of symptomatic [46] and 15–40% of asymptomatic PCR-positive patients [47,48,49], so the LFIA tests received discordant technical evaluations [47,50,51]. They had a low sensitivity in acute settings and were not suitable as a stand-alone diagnostic for acute-phase infection. However, they did identify additional COVID-19 cases among suspected patients [47].

The LFIA test was unable to detect early-stage infection [47] since antibodies take several days to reach detectable levels after contact with the virus [52]. Participants experiencing symptom onset earlier than 8–15 days before blood sampling might have had a false negative serological result [31,32,33,34,47]. However, this immunoassay is suitable for investigating individuals who present late after symptom onset and had a negative RT-PCR test (if a test were even available), assessing a population’s past or recent seroprevalence of SARS-CoV-2 infection (e.g., mild if any COVID-19 symptoms) [53], and conducting epidemiologic studies after symptom onset [31,47]. Because of the longitudinal design of our study, an infected participant with a negative result at baseline (M0) was likely to have a positive result at the three-month follow-up (M3), a sufficient time interval that results in a quasi-perfect sensitivity (100%, 95% CI: 95.5–100.0%, 15 days after symptom onset) of the serological test used in our study [31]. Because we considered participants with any positive result at M0 and M3 to be COVID+, they were recorded as COVID+ in our data.

The self-reported RT-PCR test was performed as part of a general screening practice, leading to a substantial number of under-detected cases [42]. Only one in five study participants was tested. Potential reasons for that small proportion may include the lack of testing resources during the first wave, the national health system’s limited testing capacity, or the limited implementation of the test–trace–isolate strategy.

## 5. Conclusions

The combination of symptoms (e.g., anosmia, anorexia, fever, headache, and rhinorrhea) accurately identified cancer patients with COVID-19. Specifically, our results demonstrated that some symptoms, such as headache, dysgeusia, and ageusia, had completely different expression between cancer and cancer-free populations. Accurately predicting COVID-19 from identified symptoms in cancer patients would be helpful for the diagnosis, screening, and prevention of SARS-CoV-2 infection.

## Figures and Tables

**Table 1:** Characteristics of cancer patients.

Characteristics	Cancer Patients N (%)
	N = 878
Sex	
Male	275 (31.3)
Female	603 (68.7)
Age	
Median (Range)	62 (18–91)
18–49	171 (19.5)
50–64	334 (38.0)
65–74	264 (30.1)
≥75	109 (12.4)
BMI	
Median (Range)	25 (17–43)
Obesity (BMI ≥ 30)	141 (19.9)
missing data	170
Tobacco smoking status	
Non-smoker	299 (47.8)
Former smoker	228 (36.4)
Current smoker	99 (15.8)
missing data	252
Public-facing role ^1^	
No	283 (58.1)
Yes	204 (41.9)
missing data	391
No. of Comorbidities ^2^	
≥1	330 (40.7)
missing data	67
No. of Comedications ^3^	
≥1	235 (29.0)
missing data	68
Centers of inclusion	
Nantes	201 (22.9)
Angers	238 (27.1)
Clermont-Ferrand	159 (18.1)
Nancy	280 (31.9)
Symptoms ^4^	
Symptomatic	282 (32.1)
Asymptomatic	596 (67.9)
COVID-19 tests ^5^	
Any positive test	70 (8.0)
Positive serological test	59 (6.7)
Positive RT-PCR test	26 (3.0)

^1^ Public-facing role: The question asked was: “Does your job involve contact with the public?”; ^2^ Comorbidities included: hypertension, diabetes, chronic respiratory failure, chronic kidney failure, chronic heart failure, weight loss, autoimmune disease, surgery under general anesthesia in the last 12 months; ^3^ Comedications included corticosteroids, Nonsteroidal anti-inflammatory drugs (NSAIDs), immunosuppressive drugs, and immunomodulatory drugs; ^4^ Symptomatic: having at least one COVID-19 symptom; Asymptomatic: having no COVID-19 symptoms; ^5^ Positive test results: Any positive result from M0 to M3 follow-ups.

**Table 2:** Cancer features of patients.

Cancer Features	COVID− N (%)	COVID+ N (%)	Total N (%)
	N = 808	N = 70	N = 878
Location			
Breast	335 (45)	36 (54.5)	371 (45.7)
Uterine. Endometrial. Cervical	81 (10.9)	5 (7.6)	86 (10.6)
Colorectal	32 (4.3)	3 (4.5)	35 (4.3)
Gastrointestinal	22 (3)	1 (1.5)	23 (2.8)
Prostate	59 (7.9)	0 (0)	59 (7.3)
Urological	62 (8.3)	6 (9.1)	68 (8.4)
Lung	65 (8.7)	8 (12.1)	73 (9)
Miscellaneous ^1^	89 (11.9)	7 (10.6)	96 (11.8)
missing data	63	4	67
Treatment status			
Undergoing treatment	782 (96.8)	63 (90.0)	845 (96.2)
Being monitored	26 (3.2)	7 (10.0)	33 (3.8)
Stage			
Localized	196 (27.3)	19 (31.7)	215 (27.6)
Locally advanced	122 (17.0)	9 (15.0)	131 (16.8)
Metastatic	401 (55.8)	32 (53.3)	433 (55.6)
missing data	89	10	99
ECOG PS			
0	263 (41.9)	21 (38.2)	284 (41.6)
1	333 (53.1)	31 (56.4)	364 (53.4)
≥2	31 (4.9)	3 (5.5)	34 (5)
missing data	181	15	196
Years since the first cancer diagnosis			
≥1 year	467 (62.9)	44 (66.7)	511 (63.2)
missing data	65	4	69
Last treatment before inclusion			
Chemotherapy	425 (58.7)	37 (57.8)	462 (58.6)
Immunotherapy	113 (15.6)	10 (15.6)	123 (15.6)
Targeted therapy	139 (19.2)	16 (25.0)	155 (19.7)
Hormone therapy	88 (12.2)	7 (10.9)	95 (12.1)
Radiotherapy	41 (5.7)	2 (3.1)	43 (5.5)
Surgery	22 (3.0)	4 (6.3)	26 (3.3)
Miscellaneous	23 (3.2)	0 (0)	23 (2.9)
missing data	67	6	73

^1^ Including the upper respiratory tract, brain, endocrine gland neoplasms, connective and soft-tissue neoplasms, skin, and unidentified cancers.

**Table 3:** Result of the backward logistic regression.

Predictors	OR (95% CI)	Wald	*p*-Value ^1^
Cancer Patients’ Full Model (N = 878)			
Anosmia	9.71 (2.99–31.57)	14.3	< 0.001
Dysgeusia/Ageusia	0.77 (0.26–2.35)	0.21	0.651
Fever	3.23 (1.54–6.78)	9.57	0.002
Headache	0.33 (0.12–0.90)	4.75	0.029
Rhinorrhea	1.98 (0.95–4.10)	3.36	0.067
Cough	0.61 (0.23–1.59)	1.02	0.313
Shortness of breath	1.34 (0.48–3.72)	0.32	0.575
Muscle pain	1.15 (0.50–2.67)	0.11	0.738
Intense fatigue	1.17 (0.46–2.99)	0.11	0.738
Anorexia	4.52 (1.69–12.09)	9.03	0.003
Red eyes	1.16 (0.35–3.91)	0.06	0.809
Digestive disorders	0.65 (0.25–1.66)	0.82	0.366
Chest pain	0.42 (0.11–1.67)	1.50	0.221
Cancer Patients’ Final Model (N = 878)			
Anosmia	7.48 (2.96–18.89)	18.12	< 0.001
Anorexia	3.82 (1.66–8.76)	9.99	0.002
Fever	3.07 (1.53–6.17)	9.90	0.002
Headache	0.30 (0.12–0.76)	6.49	0.011
Rhinorrhea	1.81 (0.93–3.51)	3.08	0.079
Healthcare Workers’ Full Model (N = 940)			
Anosmia	6.11 (2.26–16.49)	12.76	< 0.001
Dysgeusia/Ageusia	5.30 (1.96–14.34)	10.78	0.001
Fever	0.65 (0.30–1.41)	1.18	0.276
Headache	2.08 (0.96–4.48)	3.47	0.062
Rhinorrhea	0.67 (0.33–1.34)	1.29	0.256
Cough	1.26 (0.57–2.79)	0.32	0.570
Shortness of breath	0.90 (0.37–2.20)	0.05	0.825
Muscle pain	2.01 (0.91–4.41)	3.01	0.083
Intense fatigue	2.05 (0.89–4.73)	2.87	0.090
Anorexia	1.25 (0.47–3.30)	0.20	0.654
Red eyes	0.91 (0.31–2.65)	0.03	0.866
Digestive disorders	0.78 (0.38–1.60)	0.47	0.494
Chest pain	2.61 (1.07–6.35)	4.49	0.034
Healthcare Workers’ Final Model (N = 940)			
Anosmia	5.71 (2.21–14.75)	12.93	< 0.001
Dysgeusia/Ageusia	5.14 (2.01–13.14)	11.68	0.001
Muscle pain	1.75 (0.82–3.75)	2.08	0.149
Intense fatigue	1.78 (0.85–3.72)	2.34	0.126
Headache	1.88 (0.86–4.11)	2.53	0.111
Chest pain	2.42 (1.11–5.27)	4.95	0.026

^1^ Variable entry and removal threshold fixed at *p* = 0.20.

## Data Availability

The data that support the findings of this study are available on request from the corresponding author (K.Z.).

## Supplementary Materials

The following are available online at https://www.mdpi.com/article/10.3390/cancers13143389/s1, Supplementary I: Table S1. Characteristics of healthcare workers; Table S2. Cancer patient characteristics. COVID- versus COVID+; Table S3. Symptom prevalence and COVID-19 positive proportion by centers of inclusion; Table S4. Model validation; Supplementary II: Equations for calculating the estimated probability of a positive COVID-19 test outcome.
