# Supplementary material for: Anosmia but Not Ageusia as a COVID-19-Related Symptom among Cancer Patients—First Results from the PAPESCO-19 Cohort Study
Source: Cancers (Basel). 2021 Jul 6;13(14):3389. doi: 10.3390/cancers13143389 (PMC8303411; doi:10.3390/cancers13143389)
Supplement: Supplementary file 1 [file cancers-13-03389-s001.zip › cancers-1254195-supplementary.pdf]

cancers

Article

Anosmia But Not Ageusia as a COVID-19-Related Symptom among Cancer Patients—First Results from the PAPESCO-19 Cohort Study

Ke Zhou [1,*], Audrey Blanc-Lapierre [2], Valérie Seegers [2], Michèle Boisdron-Celle [3], Frédéric Bigot [4], Marianne Bourdon [1,5], Hakim Mahammedi [6], Aurélien Lambert [7], Mario Campone [8], Thierry Conroy [7], Frédérique Penault-Llorca [9], Martine M Bellanger [1,10] and Jean-Luc Raoul [8]

Supplementary I

Table S1. Characteristics of healthcare workers.

Characteristics	Healthcare workers N (%)
	N=940
Sex	
Male	177 (18.8)
Female	763 (81.2)
Age	
Median (Range)	40 (19–66)
18–49	706 (80.4)
50–64	233 (26.5)
65–74	1 (0.1)
≥75	0 (0)
BMI	
Median (Range)	23 (18–45)
Obesity (BMI>=30)	71 (7.8)
missing data	33
Tobacco smoking status	
Non-smoker	672 (73.6)
Former smoker	93 (10.2)
Current smoker	149 (16.3)
missing data	26
Public-facing role [1]	
No	177 (19.3)
Yes	740 (80.7)
missing data	23
No. of Comorbidities [2]	
≥1	123 (13.1)
missing data	0 (N/A)
No. of Comedications [3]	
≥1	206 (21.9)
missing data	0 (N/A)
Centers of inclusion	
Nantes	307 (32.7)
Angers	240 (25.5)
Clermont-Ferrand	344 (36.6)
Nancy	49 (5.2)
Symptoms [4]	
Symptomatic	485 (51.6)
Asymptomatic	455 (48.4)
COVID-19 tests [5]	

Any positive test	89 (9.5)
Positive serological test	73 (7.8)
Positive RT-PCR test	51 (5.4)

[1] Public-facing role: The question asked was: "Does your job involve contact with patients?"; [2] Comorbidities included: Hypertension, Diabetes, Chronic respiratory failure, Chronic kidney failure, Chronic heart failure, Weight loss, Autoimmune disease, Surgery under general anesthesia in the last twelve months; [3] Comedications included: Corticosteroids, NSAIDs (Nonsteroidal anti-inflammatory drugs), Immunosuppressive drugs, and Immunomodulatory drugs; [4] Symptomatic: Having at least one COVID-19 symptom; Asymptomatic: Having no COVID-19 symptoms; [5] Positive test results: Any positive result from M0 to M3 follow-ups.

Table S2, Part A. Cancer patient characteristics, COVID- versus COVID+, N (row % / column %).

	Patients			
	All	COVID-	COVID+	p-value
All	878 (100 / 100)	808 (92 / 100)	70 (8 / 100)	
Gender				0.04
Male	275 (100 / 31.3)	261 (94.9 / 32.3)	14 (5.1 / 20)	
Female	603 (100 / 68.7)	547 (90.7 / 67.7)	56 (9.3 / 80)	
Age				0.84
Median (Range)	60 (18-86)	60 (18-86)	61 (27-81)	
BMI				0.98
Median (Range)	25 (15-43)	25 (15-43)	25 (15-38)	
Obesity				0.67
Non-obese	737 (100 / 83.9)	680 (92.3 / 84.2)	57 (7.7 / 81.4)	
Obese	141 (100 / 16.1)	128 (90.8 / 15.8)	13 (9.2 / 18.6)	
Tobacco smoking status				0.45
Non-smoker	299 (100 / 47.8)	271 (90.6 / 47.4)	28 (9.4 / 51.9)	0.53
Former smoker	228 (100 / 36.4)	213 (93.4 / 37.2)	15 (6.6 / 27.8)	0.16
Fumeur	99 (100 / 15.8)	88 (88.9 / 15.4)	11 (11.1 / 20.4)	0.35
missing data	252	236	16	
Public-facing role [1]				0.06
No	283 (100 / 58.1)	267 (94.3 / 59.3)	16 (5.7 / 43.2)	
Yes	204 (100 / 41.9)	183 (89.7 / 40.7)	21 (10.3 / 56.8)	
missing data	391	358	33	
No. of Comorbidities [2]				0.78
≥1	330 (100 / 40.7)	300 (90.9 / 40.3)	30 (9.1 / 45.5)	
missing data	67	63	4	
No. of Comedications [3]				0.39
≥1	235 (100 / 29)	216 (91.9 / 29)	19 (8.1 / 29.2)	
missing data	68	63	5	
Centre				0.65
ICO RG Nantes	201 (100 / 22.9)	187 (93 / 23.1)	14 (7 / 20)	0.54
ICO PP Angers	238 (100 / 27.1)	218 (91.6 / 27)	20 (8.4 / 28.6)	0.76
CJP Cl.Ferrand	159 (100 / 18.1)	149 (93.7 / 18.4)	10 (6.3 / 14.3)	0.37
ICL Nancy	280 (100 / 31.9)	254 (90.7 / 31.4)	26 (9.3 / 37.1)	0.33
Cancer Location				0.94
Breast	371 (100 / 45.7)	335 (90.3 / 45)	36 (9.7 / 54.5)	0.14
Uterine. Endometrial. Cervical	86 (100 / 10.6)	81 (94.2 / 10.9)	5 (5.8 / 7.6)	0.38
Colorectal	35 (100 / 4.3)	32 (91.4 / 4.3)	3 (8.6 / 4.5)	0.92
Gastrointestinal	23 (100 / 2.8)	22 (95.7 / 3)	1 (4.3 / 1.5)	0.46
Prostate	59 (100 / 7.3)	59 (100 / 7.9)	0 (0 / 0)	-
Urological	68 (100 / 8.4)	62 (91.2 / 8.3)	6 (8.8 / 9.1)	0.83
Lung	73 (100 / 9)	65 (89 / 8.7)	8 (11 / 12.1)	0.38
Skin	23 (100 / 2.8)	21 (91.3 / 2.8)	2 (8.7 / 3)	0.92
Miscellaneous [4]	73 (100 / 9)	68 (93.2 / 9.1)	5 (6.8 / 7.6)	0.67

missing data	67	63	4	
Treatment status				0.01
Undergoing treatment	845 (100 / 96.2)	782 (92.5 / 96.8)	63 (7.5 / 90)	
Being monitored	33 (100 / 3.8)	26 (78.8 / 3.2)	7 (21.2 / 10)	

Table S2, Part B. Patient characteristics, COVID- versus COVID+, N (row % / column %).

	Patients			
	All	COVID-	COVID+	p-value
All	878 (100 / 100)	808 (92 / 100)	70 (8 / 100)	
Cancer stage				0.75
Localized	215 (100 / 27.6)	196 (91.2 / 27.3)	19 (8.8 / 31.7)	0.47
Locally advanced	131 (100 / 16.8)	122 (93.1 / 17)	9 (6.9 / 15)	0.69
Metastatic	433 (100 / 55.6)	401 (92.6 / 55.8)	32 (7.4 / 53.3)	0.72
missing data	99	89	10	
ECOG				0.41
0	284 (100 / 41.6)	263 (92.6 / 41.9)	21 (7.4 / 38.2)	0.59
1	364 (100 / 53.4)	333 (91.5 / 53.1)	31 (8.5 / 56.4)	0.64
≥2	34 (100 / 5)	31 (91.2 / 4.9)	3 (8.8 / 5.5)	0.91
missing data	196	181	15	
Years since the first cancer diagnostic				0.6
<1 year	299 (100 / 36.9)	277 (92.6 / 37.2)	22 (7.4 / 33.3)	
≥1 year	512 (100 / 63.1)	468 (91.4 / 62.8)	44 (8.6 / 66.7)	
missing data	67	63	4	
Last treatment before inclusion				
Chemotherapy	462 (100 / 57.4)	425 (92 / 57.4)	37 (8 / 57.8)	0.94
Immunotherapy	123 (100 / 15.3)	113 (91.9 / 15.2)	10 (8.1 / 15.6)	0.94
Targeted therapy	155 (100 / 19.3)	139 (89.7 / 18.8)	16 (10.3 / 25)	0.24
Hormone therapy	95 (100 / 11.8)	88 (92.6 / 11.9)	7 (7.4 / 10.9)	0.82
Radiotherapy	43 (100 / 5.3)	41 (95.3 / 5.5)	2 (4.7 / 3.1)	0.38
Surgery	26 (100 / 3.2)	22 (84.6 / 3)	4 (15.4 / 6.3)	0.2
missing data	73	67	6	
Comorbidities, detailed				
Hypertension	234 (100 / 28.9)	216 (92.3 / 29)	18 (7.7 / 27.3)	0.53
Diabetes	62 (100 / 7.6)	56 (90.3 / 7.5)	6 (9.7 / 9.1)	0.76
Respiratory Failure	54 (100 / 6.7)	46 (85.2 / 6.2)	8 (14.8 / 12.1)	0.12
Kidney Failure	35 (100 / 4.3)	28 (80 / 3.8)	7 (20 / 10.6)	0.03
Heart Failure	41 (100 / 5.1)	39 (95.1 / 5.2)	2 (4.9 / 3)	0.36
Autoimmune Disease	20 (100 / 2.5)	18 (90 / 2.4)	2 (10 / 3)	0.81
Major Surgery	186 (100 / 22.9)	167 (89.8 / 22.4)	19 (10.2 / 28.8)	0.31
missing data	67	63	4	
Comorbidities, Weight Loss				0.45
No Weight Loss	553 (100 / 68.2)	506 (91.5 / 67.9)	47 (8.5 / 71.2)	0.96
≤ 5 % during 1 mth. or ≤ 10 % during 6 mths.	54 (100 / 6.7)	51 (94.4 / 6.8)	3 (5.6 / 4.5)	0.39
≤ 10% in one mth. or ≤ 15% during 6 mths.	10 (100 / 1.2)	9 (90 / 1.2)	1 (10 / 1.5)	0.87
>5% since more than 6 mths.	8 (100 / 1)	6 (75 / 0.8)	2 (25 / 3)	0.16
missing data	67	63	4	

Table S2, Part C. Patient characteristics, COVID- versus COVID+, N (row % / column %).

	Patients			
	All	COVID-	COVID+	p-value
All	878 (100 / 100)	808 (92 / 100)	70 (8 / 100)	
Comedications, detailed				
Cortisols	210 (100 / 25.9)	191 (91 / 25.6)	19 (9 / 29.2)	0.03
NSAIDs	31 (100 / 3.8)	31 (100 / 4.2)	0 (0 / 0)	-
Immunosuppressants	5 (100 / 0.6)	5 (100 / 0.7)	0 (0 / 0)	-
Immunomodulators	3 (100 / 0.4)	2 (66.7 / 0.3)	1 (33.3 / 1.5)	0.12
missing data	68	63	5	
Symptom Onset				<0.01
Before March 17, 2020	62 (100 / 32.3)	55 (88.7 / 34.6)	7 (11.3 / 21.2)	0.12
March 17 - May 11, 2020	32 (100 / 16.7)	23 (71.9 / 14.5)	9 (28.1 / 27.3)	0.08
May 11 - October 1, 2020	68 (100 / 35.4)	62 (91.2 / 39)	6 (8.8 / 18.2)	0.02
October 1 - 30 November, 2020	30 (100 / 15.6)	19 (63.3 / 11.9)	11 (36.7 / 33.3)	<0.01
missing data	686	649	37	

[1] Public-facing role: The question asked to patients was: "Does your job involve contact with the public?"; [2] Comorbidities included: Hypertension, Diabetes, Chronic respiratory failure, Chronic kidney failure, Chronic heart failure, Weight loss, Autoimmune disease, Surgery under general anesthesia in the last twelve months; [3] Comedications included: Corticosteroids, NSAIDs (Nonsteroidal anti-inflammatory drugs), Immunosuppressive drugs, and Immunomodulatory drugs; [4] Miscellaneous included: Upper Respiratory Tract, Brain, Endocrine Gland Neoplasms, Connective and Soft Tissue Neoplasms, and unidentified cancers.

Table S3. Symptom prevalence and COVID-19 positive proportion by centers of inclusion.

	Nantes	Angers	Clermont-Fd.	Nancy	Total	p-value
Patients, Total*	201 (22.9)	238 (27.1)	159 (18.1)	280 (31.9)	878 (100)	
Symptomatic	93 (46.3)	93 (39.1)	51 (32.1)	45 (16.1)	282 (32.1)	<0.001
Asymptomatic	108 (53.7)	145 (60.9)	108 (67.9)	235 (83.9)	596 (67.9)	
COVID-	187 (93.0)	218 (91.6)	149 (93.7)	254 (90.7)	808 (92.0)	0.657
COVID+	14 (7.0)	20 (8.4)	10 (6.3)	26 (9.3)	70 (8.0)	
Age Median (Range)	61 (34–85)	63 (28–89)	64 (23–91)	63 (18–88)	62 (18–91)	0.345
HCWs, Total*	307 (32.7)	240 (25.5)	344 (36.6)	49 (5.2)	940 (100)	
Symptomatic	168 (54.7)	107 (44.6)	190 (55.2)	20 (40.8)	485 (51.6)	0.018
Asymptomatic	139 (45.3)	133 (55.4)	154 (44.8)	29 (59.2)	455 (48.4)	
COVID-	271 (88.3)	228 (95.0)	310 (90.1)	42 (85.7)	851 (90.5)	0.032
COVID+	36 (11.7)	12 (5.0)	34 (9.9)	7 (14.3)	89 (9.5)	
Age Median (Range)	40 (19–63)	40 (22–64)	41 (19–66)	46 (26–60)	40 (19–66)	0.155

*N (Horizontal %) for Total, n (Vertical %) for others.

Table S4. Model validation.

Stratified subpopulations	N	Sensitivity	Specificity	Accuracy	C-statistic
**Cancer Patient Model**					
Original dataset	878	0.1571	0.9950	0.9282	0.7027
Man only	275	0.1429	1.0000	0.9540	0.7121
Woman only	603	0.1786	0.9945	0.9187	0.7150
Age ≤ median (62 yrs.)	440	0.1579	0.9950	0.9227	0.7255
Age > median (62 yrs.)	438	0.2188	0.9951	0.9384	0.6817
Breast and Uterine/Endometrial/Cervical cancers	457	0.1463	0.9952	0.919	0.6769
Colorectal and Gastrointestinal cancer	58	0.5	1	0.9655	0.713
Miscellaneous [1]	363	0.12	0.997	0.9366	0.7214
Metastatic	443	0.1875	0.9975	0.9376	0.7472
Localized	215	0.1579	1	0.9256	0.6563
Localized advanced	131	0.3333	1	0.9542	0.7901
Chemotherapy	462	0.2162	0.9929	0.9307	0.7184
Targeted therapy	155	0.3750	1.0000	0.9355	0.7723
Hormonal therapy	95	0.2857	0.9886	0.9368	0.6916
Miscellaneous [2]	280	0.0455	0.9961	0.9214	0.7282
75% dataset	662	0.1455	0.9951	0.9245	0.6725
25% dataset	216	0.4667	0.9851	0.9491	0.7511
Nantes+Angers	439	0.2059	0.9951	0.9339	0.7352
Clermont-F.+Nancy	439	0.1389	0.9950	0.9248	0.6964
**Healthcare Worker Model**					
Original dataset	940	0.5506	0.9824	0.9415	0.8830
Man only	177	0.4706	0.9813	0.9322	0.7989
Woman only	763	0.5417	0.9783	0.9371	0.9102
Age ≤ median (40 yrs.)	477	0.5641	0.9772	0.9434	0.9059
Age > median (40 yrs.)	463	0.5000	0.9806	0.9287	0.8746
75% dataset	704	0.5313	0.9828	0.9418	0.9125
25% dataset	236	0.5600	0.9763	0.9322	0.8209
Nantes+Angers	547	0.4167	0.9840	0.9342	0.8580
Clermont-F.+Nancy	393	0.6341	0.9858	0.9491	0.9241

When there were insufficient covariate variations for logistic regression in one cancer type or cancer treatment subpopulation, two or more pertinent groups were combined.

[1] Miscellaneous cancer locations including: Prostate, Urological, Lung, Upper Respiratory Tract, Brain, Endocrine Gland Neoplasms, Connective and Soft Tissue Neoplasms, Skin, Unidentified, Others cancers, and patients with missing data on cancer location.

[2] Miscellaneous cancer treatments including: Immunotherapy, Radiotherapy, Surgery, Percutaneous ablation techniques, other treatments, and patients missing data on treatment type

Supplementary II

Equations for calculating the estimated probability of a positive COVID-19 test outcome

Patient model:

$$\hat{g}(x) = -2.97109 + 2.01222*(Anosmia=1/0) + 1.33957*(Anorexia=1/0) + 1.12181*(Fever=1/0) + (-1.20212)*(Headache=1/0) + 0.59343*(Rhinorrhea=1/0) \quad (1)$$

$$Prob.\ COVID+ = e^{\hat{g}}/(1+ e^{\hat{g}}) \quad (2)$$

HCW model:

$$\hat{g}(x) = -3.93836+1.74178*(Anosmia=1/0)+1.63699*(Dysgeusia/Ageusia=1/0)+0.56009*(Muscle\ Pain=1/0)+0.57615*(Headache=1/0)+0.63343*(Intense\ Fatigue=1/0)+0.88400*(Chest\ Pain=1/0) \quad (3)$$

$$Prob.\ COVID+ = e^{\hat{g}}/(1+ e^{\hat{g}}) \quad (4)$$
